# Familiarity, age, weaning and health status impact social proximity networks in dairy calves

**DOI:** 10.1038/s41598-023-29309-1

**Published:** 2023-02-08

**Authors:** Jorge A. Vázquez-Diosdado, Francesca Occhiuto, Charles Carslake, Jasmeet Kaler

**Affiliations:** School of Veterinary Medicine and Science, University of Nottingham, Sutton Bonington Campus, Leicestershire, LE12 5RD UK

**Keywords:** Animal behaviour, Behavioural ecology

## Abstract

Social network analysis in dairy calves has not been widely studied, with previous studies limited by the short study duration, and low number of animals and replicates. In this study, we investigated social proximity interactions of 79 Holstein–Friesian calves from 5 cohorts for up to 76 days. Networks were computed using 4-day aggregated associations obtained from ultrawideband location sensor technology, at 1 Hz sampling rate. The effect of age, familiarity, health, and weaning status on the social proximity networks of dairy calves was assessed. Networks were poorly correlated (non-stable) between the different 4-day periods, in the majority of them calves associated heterogeneously, and individuals assorted based on previous familiarity for the whole duration of the study. Age significantly increased association strength, social time and eigenvector centrality and significantly decreased closeness and coefficient of variation in association (CV). Sick calves had a significantly lower strength, social time, centrality and CV, and significantly higher closeness compared to the healthy calves. During and after weaning, calves had significantly lower closeness and CV, and significantly higher association strength, social time, and eigenvector centrality. These results indicate that age, familiarity, weaning, and sickness have a significant impact on the variation of social proximity interaction of calves.

## Introduction

Social relationships are one of the most important aspects of an animal’s life^1^, and can potentially shape individual differences in behaviour within a group^2^. Despite their relevance, social interactions, including the mechanisms, causes and consequences of individual variation in sociality, remain poorly understood^3^. Social behaviour in animals can be significantly impacted by several different factors with potential long-lasting effects^4^. For example, social conditions in early life can impact animal behaviour^5^, personality^6^, response to stress, copying behaviour^7^, cognitive development^8^ and susceptibility to disease^9^. Such effects can even be passed down to the next generation^10^.

Social network analysis (SNA) is a powerful tool^11^ that enables the understanding of different aspects of animal sociality, ranging from their underlying mechanistic process^12^ to ecological^13^ and evolutionary functions^14^. In the social network framework, nodes represent the individual animals and edges represent the relationship between pairs of individuals^15–17^. Node measures (association strength, eigenvector centrality, etc.) enable the assessment of individual social heterogeneity and help to calculate the level of connectivity of an individual^15,16^. Recent methodological advances in SNA^16,18^ and automated monitoring techniques^19^ have allowed researchers to investigate social interactions at high spatio-temporal resolutions^20^. In wild animals SNA has revealed several individual phenotypic attributes and environmental factors shaping the variability in social interactions such as age^12^, sex^13^, familiarity^21^, personality^22^ and pathogens^23^ among other.

SNA in farm animals overall has not been widely studied^17^ with only a few studies in cows^20,22,24–29^ and even fewer in calves^30–32^. In particular in calves, it has been shown that social networks vary according to different factors such as familiarity^31^, age^30,33^, and sex^30^. Results obtained in previous studies have been limited by small sample sizes, short study duration, and low number of replicates^30–32^. Regarding familiarity, Bolt et al.^31^ showed that calves that had been pair-housed spent more time with ex-penmates (familiar calves) once they were mixed into larger groups, but the time spent with ex-penmates decreased over time, suggesting the effect can eventually become insignificant. However, a previous study by Raussi et al.^33^, suggested preferences persist into adulthood, contradicting results from Bolt et al.^31^. This highlights the importance of longer studies to assess the impact of familiarity on social networks in calves. Age is known to impact the sociality of calves, as younger animals tend to be more in proximity to other calves^33^ and have lower probabilities of contact between calves with larger differences in age^30^. However, the impact that age has on other social measures^15^ (e.g. centrality), which is relevant to understand aspects such as social support, remains unknown. Additionally, results on social network stability of calves remains contradictory, with one study by Bolt et al.^31^ obtaining high significant stability between weeks in a 4-week study, and another study by Koene and Ipema^32^ finding low non-significant stability in a 10-day study. Other relevant factors in the sociality of calves, such as weaning, and health status are yet to be fully explored. Weaning is an important period in calves, as it has been shown to be a particularly stressful time^34^. Similarly, health is an important factor that has been shown to effect sociality in various species^35,36^.

The overall aim of this study was to investigate sociality in calves and its relationship with some of the most relevant factors that can affect its heterogeneity (variation in social behaviour). For this purpose, we constructed 4-day aggregated social networks from social proximity interactions of 79 calves from 5 cohorts monitored for up to 76 days. Data was collected using ultrawideband location sensor technology at 1 Hz sampling frequency, weaning stage was recorded and calves were continuously monitored and assessed for their health. The study investigated the following objectives:

1. Evaluate the stability of social proximity interactions at the group (cohort) level during the period of the study and investigate if calves associate heterogeneously in each of the social proximity networks for all the cohorts.
2. Investigate if social associations are affected by difference in age, familiarity, and differences in health status.
3. Examine the effect of factors such as age, stage of weaning and health status on social measures at the node level (strength, social time, closeness, centrality and coefficient of variation in association strength).

## Materials and methods

The present study was reviewed and approved by the Ethical Committee at the School of Veterinary Medicine and Science, University of Nottingham (Approval Number 1481 150603). All experiments were performed in accordance with relevant guidelines and regulations of the School of Veterinary Medicine and Science, University of Nottingham.

### Data collection and processing

#### Animals, housing and farm management

**Figure 1:**
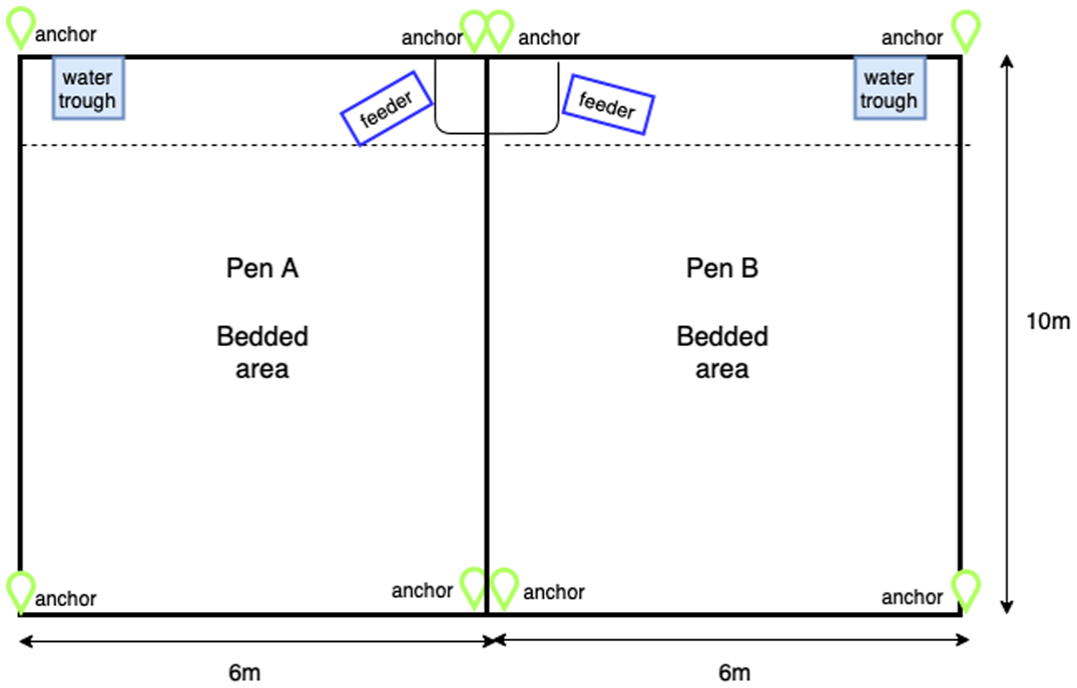
Diagram of the two pens used during this study, indicating the position of anchor sensors, the feeder and water trough areas.

The study took place at the Centre for Dairy Science Innovation at the University of Nottingham, UK, between 14th of May 2021 and 26th of November 2021. In total 79 calves from 5 different cohorts of 15 to 16 calves each were included in the study, as described in Table 1. All calves were female (F), Holstein Friesian (HF). At birth, animals were kept in pairs as per normal farm management until the calves were at least 2 weeks of age and a cohort was formed with a minimum of 15 calves. Each cohort of calves was moved to one of two adjacent straw-bedded pens as illustrated in Fig. 1. Each rectangular pen measured 6 m × 10 m, had an automatic feeder, a tank with concentrates and water trough. The feeder zone in Fig. 1 represented an area of 1.5 m × 3 m around the automatic feeder. Calves had access to concentrates and water ad-libitum. Ethical permission for all the observational procedures was obtained for the School of Veterinary Medicine and Science, University of Nottingham (unique reference number 1481 150603).

**Table 1:** Characteristics of each cohort for this study with number of animals per cohort, mean age (in days) at entry and pen location. Mean age was rounded to the nearest integer.

Cohort	Number of animals	Mean age (in days) at entry	Pen
1	16	46	B
2	15	35	A
3	16	30	B
4	16	41	A
5	16	37	B

#### Weaning and health monitoring

In this study all calves were fed milk replacer (Milkivit Energizer ECM, Trouw Nutrition GB) from an automatic feeder for the entire milk-feeding period. During the study calves were equipped with RFID ear tags that enabled their identity to be recognised by the feeding station. The automatic feeder distributed a maximum of 2 L every 2 h, up to a total daily allowance of 10 L daily for the first 35 days. From day 36 the allowance was reduced by 400 ml/day until reduced to zero on day 60. Besides their daily milk allowance, calves had also ad libitum access to concentrates (FiMLAC Sweet Start Pellets), chopped straw and water.

#### Health monitoring and sick definition

Calves were health monitored for bovine respiratory diseases (BRD), by an experienced veterinary surgeon who manually inspected all calves twice a week for signs of ill health using a modified version of the Wisconsin-Madison calf health scoring system^37^. This health assessment included clinical examination of nasal discharge, ear score, eye score and rectal temperature score. Each of these categories ranged from 0 to 3, where 0 = normal, 1 = mildly abnormal, 2 = moderately abnormal, and 3 = severely abnormal. The total health score was the sum of all the different individual scores. As per the recommendations of the scoring system^37^, calves with a total score from 0 to 2 considered were considered as being normal (healthy), scores 3 and 4 considered as moderate and values of more or equal to 5 as being sick. Bovine respiratory disease is the most common cause of sickness in pre-weaned calves^38^. There was no evidence of any other infections as per clinical exam. When assessed for health, animals showing signs of ill health were treated according to farm protocols. All calves in the study were vaccinated at 9 days of age with a respiratory vaccine (Rispoval RS + Pi3 IntraNasal; Zoetis).

#### Location position system

The location data was collected using collar mounted sensors fitted to each calf. The collars had a counterweight to help maintain the position of the sensors and were fitted 2 to 3 weeks before the calves were moved into the trial pens to allow time for habituation.

Each individual indoor tracking sensor (Sewio Leonardo iMU tags) provided relative local coordinates in (*x,y*) using ultrawideband sensor technology^39^ at a range of different frequencies. For this study, location data was collected continuously over the duration of each cohort using a sampling frequency of 1 Hz. Local position within this system was computed using distance triangulation from 4 fixed anchors located at the corners of the two adjacent pens as shown in Fig. 1.

A validation test on the accuracy of the location data was performed before the start of the study using 20 sensors at 9 static positions in the pen in a similar manner to^40^. The mean circular error probability (CEP) and a measure of the accuracy (DIST) were computed on the validation test. The CEP represents the precision of the location, and it is calculated as the radius of the circle, centred at the mean location, with 50% of the points lying within it. DIST represents the mean distance between the known ground truth location and the location obtained by the sensor. In the validation trial, the mean circular error probability (CEP) was 0.15 m (range 0.12–0.28 m) and DIST was found to be 0.17 m (range 0.13–0.33 m).

#### Pre-processing and cleaning of positional data

The location data from the 1st day when calves were moved into the pens were removed. Additionally, times when there were human visits to the pen were removed since the proximity of the calves might have been affected at these times. Times when sensors were replaced due to battery failure were also removed from the dataset as well as times when location coordinates were placed outside of the pens due to location error. In total 1.55% (5 days of the 323 total) of the data was removed from the 5 cohorts. Additionally, sensor location data was smoothed using a simple moving average over a 10-s window to improve accuracy.

#### Determining proximity interactions and social network construction

Social interactions between animals were defined as proximity interactions below a threshold distance for a minimum duration of time between a pair of calves (dyads). Such proximity interactions were non-directed (calf A being close to calf B results in calf B being close to calf A). The selection of both space and temporal thresholds was based on observations of biologically meaningful social interactions as well as the minimum distance between calves due to their size. In this study, we used a distance of 1 m and duration of 3 min for the spatial and temporal thresholds, respectively. We tested a range of different thresholds for both space (0.8 and 1.2 m) and time (2 and 4 min) and qualitatively similar results were obtained. Pair wise proximity interactions were utilised to compute edge weights (association) between individual nodes (calves). The following association index: (${{{E}}}_{{{A}}{{B}}}=\frac{{{X}}}{{{X}}+{{{Y}}}_{{{A}}{{B}}}+{{{Y}}}_{{{A}}}+{{{Y}}}_{{{B}}}}$) was utilised to compute the edge weights between any pair of individuals when the data had missing observations (represented by missing location) as suggested in^16,18^. In the association index, *X* represents the number of interactions where both animals co-occurred, *Y*_*A*_ and *Y*_*B*_ represent the number of samples where only animal A and B, were seen respectively, and *Y*_*AB*_ is the number of times both animals were observed but not in close proximity. Non-directed, weighted association matrices representing the social proximity networks, were obtained for consecutive 4-day periods of data using association indices as described before. The selection of 4-day periods was done in order to align with the health monitoring information.

### Analysis

**Figure 2:**
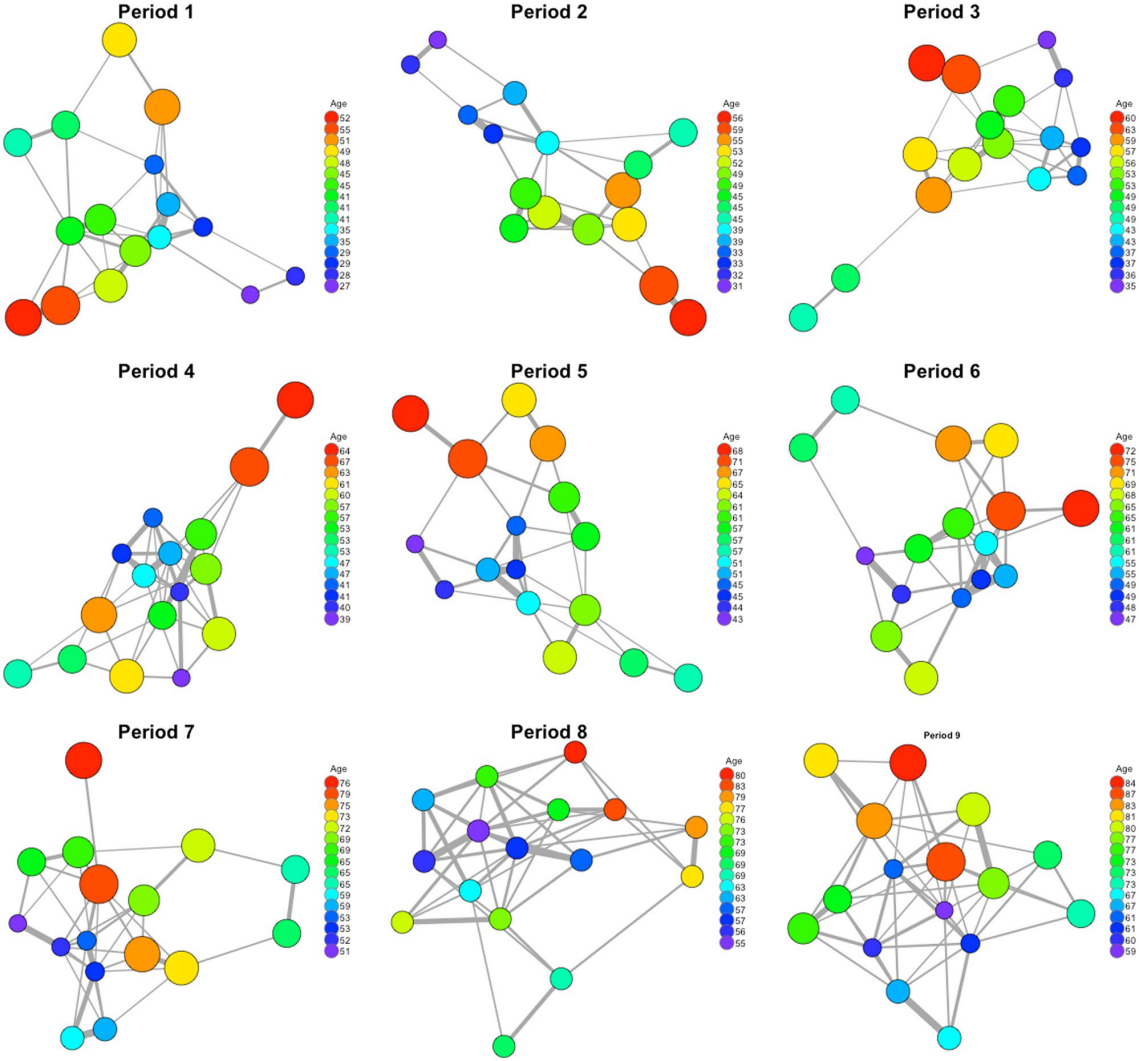
Visualisation of the networks generated for cohort 4 for the first 9 periods of 4-days. The size of the circle represents the age of the calf whereas the thickness of the line represents the edge weight (association index) between two nodes (calves).

Non-directed weighted matrices for each 4-day period for all cohorts were converted into network graphs using the package “igraph” in R^41^ to produce visualisations. In Fig. 2 we show the social network for each individual period for cohort 4. The visualisation was produced using Fruchterman–Reingold algorithm on filter networks, were edges with less than the mean were omitted.

#### Network stability (temporal variation in sociality)

Temporal variation in sociality was assessed for each cohort using all the different adjacent matrices previously obtained from association indices (edge weights) over all 4-day aggregated periods. Across all cohorts, each 4-day association matrix was compared to each other using the mantel test from the package “vegan” in R^41^ to determine the level of correlation between any two different 4-day periods. The Mantel test provided the significance of the correlation using a quadratic assignment procedure for node permutation with *n* = 10,000 for all the tests.

#### Social differentiation

We tested if the associations between calves were more heterogeneous (have higher variation) than what would be expected given the null hypothesis that they associated uniformly. For this test we used the coefficient of variation in association (edge weight) for social differentiation as computed in Withehead^18^, as the standard error over the mean where the standard error was estimated $SE= \alpha \sqrt{\frac{1-\alpha }{x}}$ where $\alpha$ is the calculated associated index and *x* is the number of samples in which A and B were interacting (proximity interaction). The original test statistic (observed coefficient of variation in association) was compared to the test statistic obtained by randomly permuting the nodes of the observed network and calculating the proportion of times when the test statistic from the permutations was equal to or more than the original test statistic. This test was performed for each individual 4-day aggregated proximity-based network on all the different cohorts using 10,000 permutations.

#### Assortment by age, health status and familiarity

Assortment measures the tendency of individuals with the same phenotypic characteristics to associate more than what would be expected if they were associating uniformly^15,16,18^. For example, older calves might interact more with other calves of similar age than with younger calves. Assortment is considered a group measure in social network analysis^15,16,18^. To test if calves associated more or less with calves of different ages, different health status, or calves they were more familiar with, we tested for assortment for each of these variables. Assortment testing was performed during the whole duration of the study on all the different 4-day periods ranging from 1 to 19 depending on how long each cohort was monitored. Familiarity was measured as the number of days they were in contact before moved into the trial pens and ranged between 0 to 51 days. The difference in age was computed between any pair of individuals as the number of days between the birth dates of the two, whereas the difference in health was computed using differences in health status with categories 0, 1 and 2, representing healthy, moderate, and sick, respectively. A MCMCglmm framework^42^ with the association index (edge weight) between any pair of individuals (IDi, IDj) as an output and fixed factors (difference in age, difference in health, and familiarity), was utilised to investigate assortment. Within this framework cohort, ${ID}_{i}$ and ${ID}_{j}$ were included as random variables. The inclusion of node IDs allowed to account for multiple membership of the association as suggested by Franks et al.^43^. Edge weight (association index) was log transformed to satisfy the assumptions of normality. Similarly, the model was also assessed for heteroscedasticity by assessing residual vs fitted values. Independence was dealt using the multiple membership random effect as described in Franks^43^.

#### Impact of age, calf health and weaning on sociality measures

We investigated the impact that weaning and health have on sociality by fitting a mixed effect linear model using the lme4 package^44^ in R^41^ for each of the following social measures: total social time, association strength, eigenvector centrality, closeness, and coefficient of variation in association strength.

*Total social time* was computed as the sum of the proximity interactions for each individual calf with the rest of the group. To convert this value into minutes it was then multiplied by 3 given that proximity interactions were calculated using a 3-min threshold. This measure was computed without using association index. The rest of the social measures (association strength, eigenvector centrality, closeness, and coefficient of variation in association) were computed using the association index from pair wise proximity interactions at each individual 4-day period as follows:

*Association strength* was computed as the sum of the edge weights (association indices) for each individual calf, and it represents individuals’ sociality or social activity^15,16^.

*Eigenvector centrality* is the sum of the centralities of an individuals’ neighbours, and it is computed as the first non-negative value obtained from the adjacency matrix representation of the network. Eigenvector centrality represents the “importance” of the individual within the network^15,16^.

*Closeness centrality* is measured in terms of shortest paths between the node with all the rest of the nodes in the network and it represents its potential for disease or information transmission^45^.

*Coefficient of variation in association* is a standardised, unit free measure of dispersion which was computed as suggested by Whitehead and indicates the level of heterogeneity in associations^18^.

The models were defined as follows:

1 $$Y=X\beta +Z\alpha +\varepsilon $$

where *Y* represents each of the social measures (total social time, association strength, eigenvector centrality, closeness and coefficient of variation in association), *X* represents the fixed effects for the 4-day observational time unit: age of the calf (taken at the end of the 4-day period), weaning stage and health status of the calf, respectively. *Z* represents the random effects: Calf ID and period nested within cohort and $\varepsilon$ represents the residuals. Weaning was defined as categorical variable with three categories (non- weaned, step-down, and weaned). Health was defined as categorical variable with three categories (healthy, moderate, and sick) as previously defined. For each of the models (e.g., association strength), the outcome variable and fixed factors were obtained per individual calf. The Benjamini–Hochberg (BH) procedure^46^ was applied for multiple significant testing over the same data.

### Ethics

Ethical permission for all the methods of the observational trial described was obtained for the School of Veterinary Medicine and Science, University of Nottingham (unique reference number 1481 150603). All methods are reported in accordance with the ARRIVE guidelines^47^.

## Results

### Temporal variation in sociality

**Figure 3:**
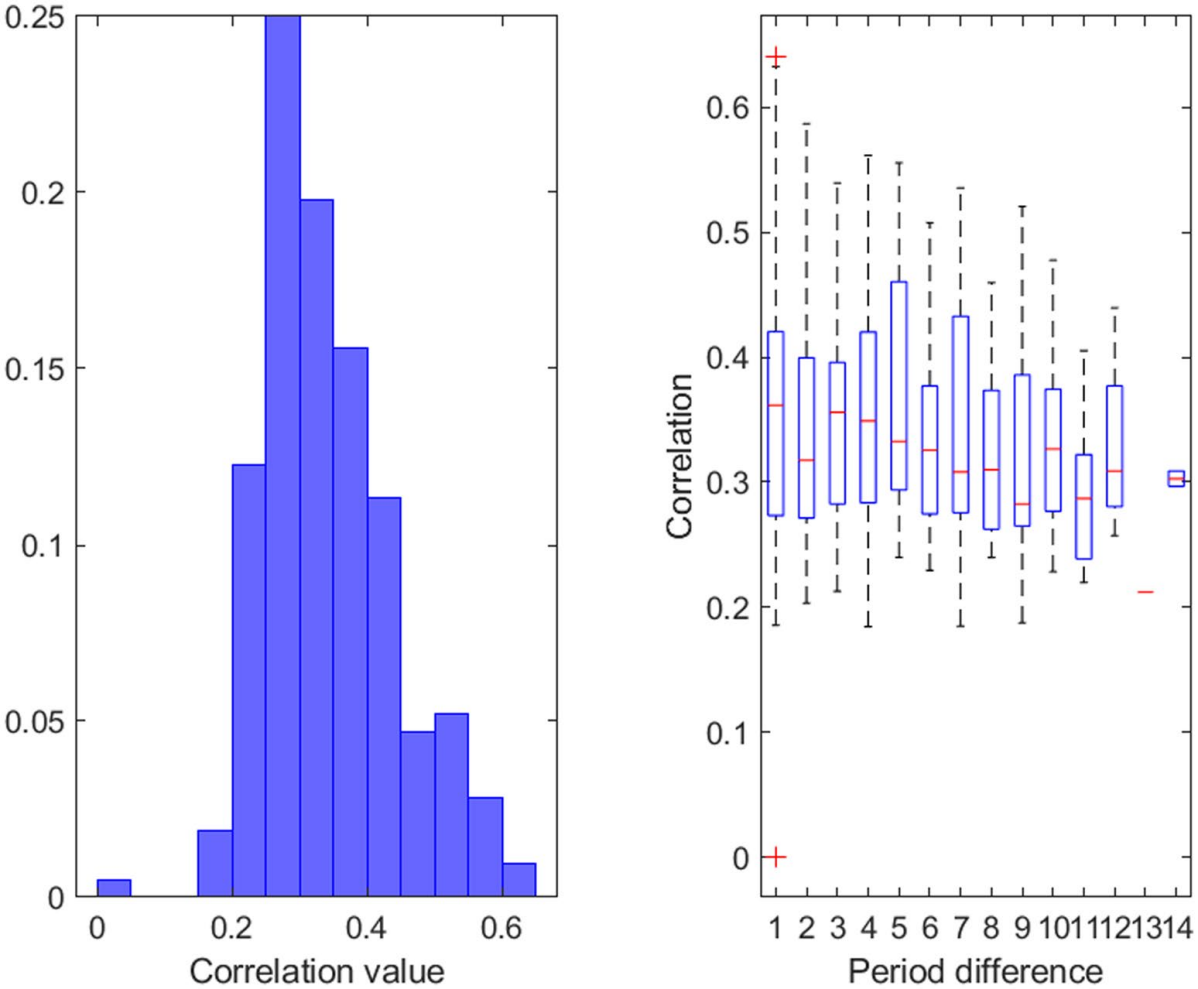
Distribution of the significant correlations between different periods (left) and boxplot of the significant correlations between different periods according to the different in time between periods (e.g. 2 represent a difference of 2 periods).

Out of all the correlations between the different periods, 41.09% were significant. Figure 3 shows the distribution of the significant correlation values between different periods as well as boxplot with the significant correlation between different periods grouped by the difference in time period (e.g. a difference of 2 can come from periods 2 and 4, 3 and 5, etc.). The significant correlations had a mean of 0.3423 (± 0.0984).

### Social differentiation

For all cohorts there was a significant social differentiation at each 4-day aggregated proximity-based network, with the exception of period 5 for cohort 1. The results of the social differentiation test are shown in the Supplementary Material (Table 1). This demonstrates that calves in general calves associated heterogeneously, expending more time with certain individuals than with others.

### Assortment by age, health status and familiarity

There was significant assortment by familiarity in all periods for all cohorts, as calves spent significantly more time with other calves that they were familiar with. There was also significant assortment by difference in age in weeks 1–2, 4–12 and 16–19, meaning that calves spent less time with calves of different ages (see Supplementary Material, Table 2). There was also significant assortment by difference in health only at period 1, indicating that calves spent less time with those with a different health status only in the first 4-day period.

**Table 2:** Effects of age, health status, and weaning status in the different association network measures. The coefficients and *p*-values are derived from the linear mixed model. In bold are the coefficient with significant values.

Factor	Strength		Time sociality		Closeness		Centrality		Coefficient of Variation	
	β	p-value	Β	p-value	Β	p-value	Β	p-value	β	p-value
Intercept	0.742	< 0.001	1377	< 0.001	0.626	< 0.001	0.655	< 0.001	1.150	< 0.001
Age	− 0.003	0.002	− 4.79	0.005	0.147	< 0.001	− 0.100	< 0.001	0.037	0.061
Health moderate	− 0.007	0.385	5.45	0.7342	0.014	0.3418	− 0.011	0.336	− 0.013	0.336
Health sick	− 0.047	0.003	− 68.98	0.0200	0.101	< 0.001	− 0.056	0.005	− 0.064	0.006
Weaning step-down	0.072	< 0.001	180.74	< 0.001	− 0.129	0.001	0.087	0.033	− 0.074	0.006
Weaned	0.071	0.006	176.97	< 0.001	− 0.166	0.0012	0.184	0.003	− 0.093	0.013

### Association between age, individual health/weaning and sociality measures

The results from the linear mixed model showed that older calves had significantly lower association strength (*t* = − 6.065, *p*-value = 0.0024), significantly lower social time (*t* = − 4.331, *p*-value = 0.0031), significantly higher closeness (*t* = 6.85, *p*-value = 0.0007) and lower centrality (t = − 5.866, *p*-value = 0.0001) than younger calves (Table 2).

Sick calves had significantly higher closeness (*t* = 3.81, *p*-value = 0.0004) and significantly lower (*t* = − 3.17, *p*-value = 0.0031) association strength, social time (*t* = − 2.42, *p*-value = 0.0200), centrality (*t* = − 2.95, *p*-value = 0.0054) and coefficient of variation (*t* = − 2.86, *p*-value = 0.0061), than healthy calves.

During the step-down weaning process calves had significantly higher strength (*t* = 5.38 *p*-value = 0.0024), social time (*t* = 6.58, *p*-value = 0.0001) and centrality (*t* = 3.75, *p*-value 0.0335), and significantly lower closeness (*t* = − 5.44, *p*-value = 0.0002)and coefficient of variation (*t* = − 3.38, *p*-value = 0.0061) than pre-weaned calves. Similarly, weaned calves had significantly higher strength (*t* = 3.16 *p*-value = 0.0061), social time (*t* = 3.75, *p*-value = 0.002) and centrality (*t* = 4.86, *p*-value 0.0035), and significantly lower closeness (*t* = − 4.02, *p*-value = 0.0012), and coefficient of variation (*t* = − 2.78, *p*-value = 0.01) than calves before weaning.

## Discussion

This is the first study that investigated social networks and social proximity interactions obtained from high temporal resolution of location data in calves, and the impact of the most relevant factors on calves’ sociality. We obtained novel insights on the effect that age, weaning stage and health status have on several social metrics at the node level. We evaluated the stability of social proximity interactions and the social differentiation and investigated for the first time the effect that familiarity, difference in health and difference in age have on associated social network strength.

To the author’s knowledge, this is the first study that shows a significant effect of weaning stage and health status on social measures at the individual animal level. While previous studies have shown an increase in the number of vocalisations during and after weaning^31^, no information on social proximity encounters and social network metrics has been previously reported. Our results show that during weaning calves had higher association strength, social time and centrality, and lower closeness and coefficient of variation than pre-weaned calves. This means that during the weaning period calves were socialising more (higher number of interactions and association strength) and were more central than non-weaned calves while they were less close to other calves and had lower heterogeneity in their interactions (lower coefficient of variation in association). For example, during weaning calves had approximately 180.74 more proximity encounters of three-minute duration (Table 2), which correspond to an additional 9.03 h socialising. Weaning is known to be a stressful time for animals^34,48,49^ and calves might alleviate the stress via ‘social support’ as this has been shown to improve individuals’ abilities to cope with challenges^50^. In older cattle, it has been shown that after being exposed to a stressor event (post-handling) animals will prefer social environments that allow social interactions with peers^51^. Eigenvector centrality can be interpreted as the social support or social capital of an individual^15^ and hence higher centrality indicates higher support during this stressful time. Additionally, it has been previously shown that calves going through a painful procedure such as disbudding spend more time with other calves recovering from the procedure^52^. Once the calves were weaned, they continued to have higher association strength, social time, eigenvector centrality and lower closeness and coefficient of variation in association in our study. This might be due to the fact that weaned calves spend less time around the feeder which might allow them to spend more time socialising and thus increases their association strength, social time and their position within the network (eigenvector centrality).

Another key result of this study was the impact of health status on sociality in calves. Previous studies have only investigated if there were any significant differences in health status between different housing types^31^, with no information on the effect that sickness has on social measures. We showed that sick calves had lower association strength, social time, eigenvector centrality, and coefficient of variation in association and higher closeness. A lower coefficient of variation in association indicates that calves increase the number of non-preferential proximity encounters. Eigenvector centrality represent the importance of the animal within the group^16^ and as such it might require high levels of energy to maintain. Sick calves affected by bovine respiratory disease (BRD) have lower feed consumption^53^ (energy intakes), hence by reducing their eigenvector centrality animals might be adopting a strategy to maintain their energy balance during periods of illness^54^. Lower association strength and eigenvector centrality during sickness have been shown in other species^36^. Closeness has been used as measure of disease transmission^15,16,45^ indicating that nodes with higher values will transmit more easily diseases within the group. Changes in social behaviour might be the result of parasites manipulating the individual behaviour of the host to facilitate parasite transmission^55,56^. In our study, undirected networks were obtained from proximity interactions, and hence it was not possible to determine if sick animals actively avoided social interactions with others, or if other healthy animals avoided the sick animal or a combination of both.

Our results showed that older calves associated less than younger individuals (lower strength and social time), which is in agreement with a previous study^33^. This might be due to higher social exploration^57^ in younger calves while older calves become more solitary as they age, which has been shown in other species (i.e. giraffes^57,58^). Additionally, older calves had lower centrality (social rank), higher closeness, and higher coefficient of variation. This might be the result of animals strengthening their proximity interactions with specific individuals over time. Additionally, our results showed assortment by age difference in most of the different periods, indicating that calves associated more with others of similar age.

Our results show that assortment by familiarity was maintained through the whole duration of this study (up to the 19th period, which corresponds to 76 days), supporting previous evidence of early social relationships maintained over long periods of time^33,59^. Although this result is in agreement with previous research showing that early preferences persist for longer durations of times up to adulthood^33^, it is in contrast with Bolt et al.^31^ who found that familiarity reduced overtime. The differences in the results might be due to the duration of time investigated (4 weeks in^31^), as well as the duration of time that calves were paired before being grouped.

In the current study calves associated heterogeneously for the majority of the 4-day periods, meaning that they had more interactions with certain individuals, which is in agreement with previous studies also showing heterogeneity in social networks in dairy calves^20,31^. A higher degree of selectivity has been previously linked to pessimism in dairy calves^60^, and therefore highlights the possible link between social network measures and personality measures, for which there is emerging evidence in calves^61–63^. Additionally, the results on network stability indicate that networks were not stable all the time as only 41% of all the possible correlations between any two 4-day aggregated networks were significantly positive. When significantly correlated, all values were positive and relatively high (r = 0.3423 (± 0.0984)), and this did not depend on the time difference between the two different periods (e.g., longer the time between periods larger the correlation or vice versa). Previous studies have reported contradicting results for network stability with^20,26,32^ who also reported no temporal stability in cattle and calf networks and Bolt et al.^31^ who showed high significant correlation between all weeks in calves. Disparity between our results and those from Bolt et al.^31^ might be due to the differences in the methodology, as they have a larger group size (n = 40) in a larger pen (200 m^2^) and the differences in the preceding rearing and grouping protocols. Additionally, it has been shown that stability in social proximity patterns might depend on the activity that calves are performing when in proximity (lying and standing)^60^. Hence, network stability results obtained in this study might be different if social proximity networks were to be separated by activity, which could be explored in future work.

Our results indicate that age, familiarity, weaning and sickness have a significant importance in the variation of social proximity interaction of calves. However, other important factors such as differences in animal personality within a group can also drive collective behaviour and hence sociality in animals^1,64^. Variation in sociality can also play a role in animal personality, creating a feedback loop^1^. Future work could focus on understanding the dynamics between animal personality and sociality in calves. Moreover, sociality can also be investigated as a personality trait by quantifying the consistency of an individual’s network position over time and contexts^1,16^. One limitation of the current study is that only one type of interaction has been investigated: social proximity interactions, which are a type of affiliative interaction, without examining other affiliative (e.g., allogrooming) or agnostic interactions (e.g. displacements). It has been shown that the effect of illness on social interactions depends on the type behaviour^65^ and hence other types of interactions might display different effects. Future research should include such social behaviours which can be potentially monitored using a combination of technologies to detect and quantify them^24^.

Despite some of the limitations of the current study, our results provide new insights into the social behaviour of calves and the factors that have a significant impact on it, and hence it has potential for use on developing stress-buffering (coping) strategies for the improvement of the welfare of farm animals^50^. Some of the key strengths of this study are the use of multiple replicates (5 replicates), the long-term monitoring (up to 76 days) and the large total number of animals (76 calves) compared to previous studies^30–32^. The number of replicates has been shown to be highly important for hypothesis testing in social behaviour^66^. Moreover, the novel insights on the factors that affect and/or shape social interactions in calves can be used to develop and implement better farm practices^67^ and could lead to the development of algorithms for the automatic detection of sick animals, which would ultimately improve farm animal health and welfare.

## Supplementary Information

Supplementary Tables.

## Data Availability

The datasets supporting the results of this article are available upon reasonable request from the corresponding author (Jorge.VazquezDiosdado@nottingham.ac.uk).
