# Supplementary material for: Familiarity, age, weaning and health status impact social proximity networks in dairy calves
Source: Sci Rep. 2023 Feb 8;13:2275. doi: 10.1038/s41598-023-29309-1 (PMC9908884; doi:10.1038/s41598-023-29309-1)
Supplement: Supplementary file 1 — Supplementary Tables. [file 41598_2023_29309_MOESM1_ESM.docx]

Supplementary Material on

“Familiarity, age, weaning and health status impact in social proximity networks in dairy calves”

J. A. Vázquez-Diosdado*^1^, Francesca Occhiuto^1^, C.E.B. Carslake^1^, and J. Kaler^1^

^1^School of Veterinary Medicine and Science, University of Nottingham, Sutton Bonington Campus, Leicestershire, LE12 5RD, United Kingdom

Correspondence: Jorge.VazquezDiosdado@nottingham.ac.uk

Results of social differentiation test are shown in the following table 1.

Table 1. Social heterogeneity of calves measured at the group level using coefficient of variation (CV) in association from the observed networks vs measures with permuted networks. In bold are the significant *p*-values. In the table CV represent the coefficient of variation in association for the observed network. The p-value was computed as the proportion of times

|  | ***Cohort 1*** | | ***Cohort 2*** | | ***Cohort 3*** | | ***Cohort 4*** | | ***Cohort 5*** | |
| --- | --- | --- | --- | --- | --- | --- | --- | --- | --- | --- |
| ***Period*** | ***CV*** | ***p-value*** | ***CV*** | ***p-value*** | ***CV*** | ***p-value*** | ***CV*** | ***p-value*** | ***CV*** | ***p-value*** |
| ***1*** | 1.13 | <0.001 | 1.30 | <0.01 | 1.19 | <0.01 | 1.1686 | <0.001 | 1.16 | 0.017 |
| ***2*** | 1.11 | <0.001 | 1.21 | <0.001 | 1.15 | <0.01 | 1.1970 | <0.01 | 1.15 | <0.001 |
| ***3*** | 1.11 | <0.001 | 1.17 | <0.001 | 1.15 | <0.01 | 1.1786 | <0.01 | 1.14 | <0.001 |
| ***4*** | 1.10 | <0.001 | 1.09 | <0.001 | 1.15 | <0.001 | 1.1346 | 0.010 | 1.11 | <0.001 |
| ***5*** | 1.09 | 0.4602 | 1.11 | <0.001 | 1.13 | <0.01 | 1.1348 | <0.01 | 1.12 | <0.01 |
| ***6*** | 1.10 | <0.001 | 1.08 | <0.001 | 1.15 | <0.01 | 1.09 | <0.01 | 1.12 | 0.015 |
| ***7*** | 1.07 | <0.001 | 1.08 | <0.001 | 1.12 | <0.01 | 1.10 | 0.022 | 1.08 | <0.01 |
| ***8*** | 1.08 | <0.001 | 1.08 | <0.001 | 1.17 | <0.001 | 1.11 | <0.001 | 1.11 | <0.01 |
| ***9*** | 1.04 | <0.001 | 1.09 | <0.001 | 1.08 | <0.001 | 1.10 | <0.001 | 1.14 | <0.001 |
| ***10*** | 1.09 | <0.001 | 1.08 | <0.001 | 1.09 | <0.001 | 1.10 | <0.001 | 1.10 | <0.001 |
| ***11*** | 1.07 | <0.001 | 1.07 | <0.001 | 1.12 | <0.01 | 1.09 | <0.01 | 1.09 | <0.01 |
| ***12*** | 1.07 | <0.001 | 1.10 | <0.001 | 1.09 | <0.001 | 1.13 | <0.001 | 1.08 | <0.001 |
| ***13*** |  |  | 1.10 | <0.001 | 1.09 | <0.001 | 1.10 | <0.001 | 1.08 | <0.01 |
| ***14*** |  |  | 1.08 | <0.001 | 1.09 | <0.001 | 1.08 | <0.001 | 1.09 | <0.01 |
| ***15*** |  |  | 1.06 | <0.001 | 1.08 | <0.001 | 1.09 | <0.001 | 1.10 | <0.001 |
| ***16*** |  |  | 1.07 | <0.001 | 1.10 | <0.001 | 1.10 | <0.001 |  |  |
| ***17*** |  |  |  |  | 1.11 | <0.001 | 1.09 | <0.01 |  |  |
| ***18*** |  |  |  |  | 1.11 | <0.001 |  |  |  |  |
| ***19*** |  |  |  |  | 1.09 | <0.001 |  |  |  |  |

Table 2. Effects of difference in age, difference in health status and familiarity in association strength. Posterior mean, 95% credible interval, and *p*-value based on MCMCglmm framework. In bold are the significant *p*-values.

|  | ^Factor^ | ^Posterior Mean^ | ^I-95% CI^ | ^U-95% CI^ | ^p-value^ |
| --- | --- | --- | --- | --- | --- |
| ^Period 1^ | ^Intercept^ | -3.857 | -4.056 | -3.690 | <0.001 |
|  | ^Familiarity^ | 0.052 | 0.043 | 0.062 | <0.001 |
|  | ^Difference in Age^ | -0.015 | -0.027 | -0.002 | 0.0143 |
|  | ^Difference in Health^ | 0.193 | 0.069 | 0.341 | **0.0143** |
| ^Period 2^ | ^Intercept^ | -3.825 | -4.022 | -3.636 | <0.001 |
|  | ^Familiarity^ | 0.053 | 0.043 | 0.063 | <0.001 |
|  | ^Difference in Age^ | -0.015 | -0.027 | -0.001 | 0.02 |
|  | ^Difference in Health^ | 0.074 | -0.088 | 0.219 | 0.326 |
| ^Period 3^ | ^Intercept^ | -3.835 | -4.045 | -3.609 | <0.001 |
|  | ^Familiarity^ | 0.053 | 0.044 | 0.062 | <0.001 |
|  | ^Difference in Age^ | -0.013 | -0.025 | 0.0008 | 0.054 |
|  | ^Difference in Health^ | 0.046 | -0.081 | 0.151 | 0.437 |
| ^Period 4^ | ^Intercept^ | -3.834 | -4.033 | -3.643 | <0.001 |
|  | ^Familiarity^ | 0.053 | 0.043 | 0.062 | **<0.001** |
|  | ^Difference in Age^ | -0.015 | -0.028 | -0.002 | 0.025 |
|  | ^Difference in Health^ | 0.112 | -0.030 | 0.258 | 0.125 |
| ^Period 5^ | ^Intercept^ | -3.846 | -4.066 | -3.636 | <0.001 |
|  | ^Familiarity^ | 0.053 | 0.044 | 0.063 | <0.001 |
|  | ^Difference in Age^ | -0.013 | -0.026 | -0.0003 | 0.048 |
|  | ^Difference in Health^ | 0.180 | -0.062 | 0.376 | 0.108 |
| ^Period 6^ | ^Intercept^ | -3.776 | -3.966 | -3.591 | <0.001 |
|  | ^Familiarity^ | 0.052 | 0.042 | 0.062 | <0.001 |
|  | ^Difference in Age^ | -0.015 | -0.026 | -0.001 | 0.02 |
|  | ^Difference in Health^ | -0.069 | -0.248 | 0.100 | 0.471 |
| ^Period 7^ | ^Intercept^ | -3.799 | -4.049 | -3.555 | <0.001 |
|  | ^Familiarity^ | 0.052 | 0.043 | 0.063 | <0.001 |
|  | ^Difference in Age^ | -0.014 | -0.029 | -0.0008 | 0.04 |
|  | ^Difference in Health^ | 0.038 | -0.157 | 0.215 | 0.717 |
| ^Period 8^ | ^Intercept^ | -3.840 | -4.100 | -3.598 | <0.001 |
|  | ^Familiarity^ | 0.0526 | 0.043 | 0.062 | <0.001 |
|  | ^Difference in Age^ | -0.013 | -0.027 | -0.0001 | 0.048 |
|  | ^Difference in Health^ | 0.102 | -0.089 | 0.270 | 0.225 |
| ^Period 9^ | ^Intercept^ | -3.795 | -4.066 | -3.553 | <0.001 |
|  | ^Familiarity^ | 0.053 | 0.042 | 0.063 | <0.001 |
|  | ^Difference in Age^ | -0.015 | -0.027 | -0.001 | 0.017 |
|  | ^Difference in Health^ | 0.031 | -0.117 | 0.189 | 0.7 |
| ^Period 10^ | ^Intercept^ | -3.802 | -3.988 | -3.582 | <0.001 |
|  | ^Familiarity^ | 0.053 | 0.043 | 0.064 | <0.001 |
|  | ^Difference in Age^ | -0.015 | -0.027 | -0.0002 | 0.028 |
|  | ^Difference in Health^ | 0.034 | -0.105 | 0.193 | 0.685 |
| ^Period 11^ | ^Intercept^ | -3.764 | -3.997 | -3.540 | <0.001 |
|  | ^Familiarity^ | 0.053 | 0.044 | 0.062 | <0.001 |
|  | ^Difference in Age^ | -0.015 | -0.027 | -0.002 | 0.025 |
|  | ^Difference in Health^ | -0.054 | -0.221 | 0.103 | 0.514 |
| ^Period 12^ | ^Intercept^ | -3.848 | -4.163 | -3.578 | <0.001 |
|  | ^Familiarity^ | 0.052 | 0.043 | 0.062 | <0.001 |
|  | ^Difference in Age^ | -0.014 | -0.026 | 0.0005 | 0.037 |
|  | ^Difference in Health^ | 0.117 | -0.035 | 0.265 | 0.157 |
| ^Period 13^ |  |  |  |  |  |
|  | ^Intercept^ | -3.859 | -4.116 | -3.530 | **<0.001** |
|  | ^Familiarity^ | 0.055 | 0.043 | 0.067 | **<0.001** |
|  | ^Difference in Age^ | -0.014 | -0.030 | 0.000 | 0.051 |
|  | ^Difference in Health^ | 0.008 | -0.163 | 0.195 | 0.925 |
| ^Period 14^ |  |  |  |  |  |
|  | ^Intercept^ | -3.858 | -4.202 | -3.558 | **<0.001** |
|  | ^Familiarity^ | 0.054 | 0.043 | 0.066 | **<0.001** |
|  | ^Difference in Age^ | -0.010 | -0.028 | 0.004 | 0.203 |
|  | ^Difference in Health^ | -0.054 | -0.255 | 0.185 | 0.643 |
| ^Period 15^ |  |  |  |  |  |
|  | ^Intercept^ | -3.830 | -4.12 | -3.506 | **<0.001** |
|  | ^Familiarity^ | 0.054 | 0.043 | 0.064 | **<0.001** |
|  | ^Difference in Age^ | -0.012 | -0.028 | 0.002 | 0.12 |
|  | ^Difference in Health^ | -0.106 | -0.377 | 0.121 | 0.371 |
| ^Period 16^ |  |  |  |  |  |
|  | ^Intercept^ | -3.790 | -4.177 | -3.388 | **<0.001** |
|  | ^Familiarity^ | 0.055 | 0.043 | 0.066 | **<0.001** |
|  | ^Difference in Age^ | -0.017 | -0.036 | -0.000 | **0.048** |
|  | ^Difference in Health^ | -0.010 | -0.304 | 0.239 | 0.905 |
| ^Period 17^ |  |  |  |  |  |
|  | ^Intercept^ | -3.487 | -3.776 | -3.250 | **0.002** |
|  | ^Familiarity^ | 0.046 | 0.031 | 0.060 | **< 0.001** |
|  | ^Difference in Age^ | -0.025 | -0.044 | -0.005 | **0.002** |
|  | ^Difference in Health^ | -0.389 | -0.772 | 0.003 | 0.054 |
| ^Period 18^ |  |  |  |  |  |
|  | ^Intercept^ | -3.765 | -4.056 | -3.458 | **0.002** |
|  | ^Familiarity^ | 0.055 | 0.041 | 0.067 | **< 0.001** |
|  | ^Difference in Age^ | -0.019 | -0.036 | -0.001 | **0.04** |
|  | ^Difference in Health^ | 0.023 | -0.252 | 0.300 | 0.854 |
| ^Period 19^ |  |  |  |  |  |
|  | ^Intercept^ | -3.496 | -3.782 | -3.241 | **0.002** |
|  | ^Familiarity^ | 0.048 | 0.034 | 0.062 | **< 0.001** |
|  | ^Difference in Age^ | -0.025 | -0.043 | -0.004 | **0.02** |
|  | ^Difference in Health^ | -0.384 | -0.800 | 0.038 | 0.071 |
